# High-risk pregnancies and their association with severe maternal morbidity in Nepal: A prospective cohort study

**DOI:** 10.1371/journal.pone.0244072

**Published:** 2020-12-28

**Authors:** Sushma Rajbanshi, Mohd Noor Norhayati, Nik Hussain Nik Hazlina

**Affiliations:** 1 Women’s Health Development Unit, School of Medical Sciences, Universiti Sains Malaysia, Kubang Kerian, Kelantan, Malaysia; 2 Department of Family Medicine, School of Medical Sciences, Universiti Sains Malaysia, Kubang Kerian, Kelantan, Malaysia; University of Mississippi Medical Center, UNITED STATES

## Abstract

**Background:**

The early identification of pregnant women at risk of developing complications at birth is fundamental to antenatal care and an important strategy in preventing maternal death. This study aimed to determine the prevalence of high-risk pregnancies and explore the association between risk stratification and severe maternal morbidity.

**Methods:**

This hospital-based prospective cohort study included 346 pregnant women between 28–32 gestational weeks who were followed up after childbirth at Koshi Hospital in Nepal. The Malaysian antenatal risk stratification approach, which applies four color codes, was used: red and yellow denote high-risk women, while green and white indicate low-risk women based on maternal past and present medical and obstetric risk factors. The World Health Organization criteria were used to identify women with severe maternal morbidity. Multivariate confirmatory logistic regression analysis was performed to adjust for possible confounders (age and mode of birth) and explore the association between risk stratification and severe maternal morbidity.

**Results:**

The prevalence of high-risk pregnancies was 14.4%. Based on the color-coded risk stratification, 7.5% of the women were categorized red, 6.9% yellow, 72.0% green, and 13.6% white. The women with high-risk pregnancies were 4.2 times more likely to develop severe maternal morbidity conditions during childbirth.

**Conclusions:**

Although smaller in percentage, the chances of severe maternal morbidity among high-risk pregnancies were higher than those of low-risk pregnancies. This risk scoring approach shows the potential to predict severe maternal morbidity if routine screening is implemented at antenatal care services. Notwithstanding, unpredictable severe maternal morbidity events also occur among low-risk pregnant women, thus all pregnant women require vigilance and quality obstetrics care but high-risk pregnant women require specialized care and referral.

## Background

A high-risk pregnancy is any condition associated with a pregnancy where there is an actual or potential risk to the mother or fetus [1]. Women with risk factors for high-risk pregnancies have a one in four chance of developing complications than those with a low risk of high-risk pregnancies who have nearly one in ten [2]. The central focus of maternal and childcare programs has been detecting at-risk pregnancies to prevent women from developing obstetric complications in childbirth [3, 4]. Risk assessment is a key component of antenatal care (ANC) and has demonstrated benefits in improving maternal and perinatal outcomes [5–7].

Maternal mortality remains a major public health issue worldwide, particularly in low resource countries, which account for 85% of maternal deaths [8]. About 40%–50% of maternal deaths are deemed preventable [9]. The maternal mortality ratio in Nepal is 239 per 100,000 live births [10]. Consistent with maternal mortality, severe maternal morbidity (SMM) rates are higher in low- and middle-income countries [11]. The World Health Organization (WHO) adopted and defined standard criteria for maternal near miss (MNM) and SMM in 2009. These criteria provide common ground for the identification of MNM and SMM as well as comparisons of MNM and SMM between countries [12, 13]. Studies on maternal morbidity are widely conducted as alternative ways to monitor standards of obstetric care [14] while studying the quality of maternal care is an alternative strategy to reducing maternal mortality [15, 16].

A risk-oriented approach using color codes (red, yellow, green, and white) was adopted in Malaysia in 1989 [17, 18]. Using this approach, a woman’s risk status is assessed throughout her ANC visits, and the allocated color code may change at each visit. The color-coded function is used as a managerial tool to determine the appropriate care providers and the location of further ANC visits and childbirth. This approach is routinely practiced in Malaysia and is included in the country’s checklist guideline for health care of mother and baby following the color code system [19].

Risk scoring systems use risk factors during the antepartum, intrapartum, and neonatal periods separately or in combination for risk stratification. Studies have suggested that to manage antepartum conditions, there should be an adequate period between the identification of the risk factors and childbirth [20–22]. Obstetric complications may occur anytime during pregnancy, labor, birth, and puerperium, ranging from mild to severe, sometimes life-threatening. The most accurate estimates of at–risks women can be made during late periods of pregnancy [23].

To prevent SMM, it is imperative to pay attention to those women at highest risk [9]. Risk assessment tools are only of value if it can prevent adverse effects in both mothers and their newborns. Risk assessment tools look at risk factors comprehensively, making it harder for risk factors to be overlooked. The purpose of this study was to determine (i) the prevalence of high-risk pregnancies and (ii) the association between risk stratification and SMM. In this study, high-risk pregnancies referred to women with red and yellow codes, while women with green and white codes were considered low-risk based on the Malaysian antenatal risk stratification. This color-coded risk stratification was mainly used because it can be applied at any time during pregnancy.

Additionally, it is easier to use, which does not include scoring and its summation. Malaysia has adopted this approach at a national level. This risk approach strategy, along with other interventions, has been credited with successfully reducing maternal morbidity to 28 per 100,000 live births in Malaysia [16]. In this study, SMM referred to “potentially life-threatening conditions during pregnancy, childbirth or after the termination of pregnancy from which maternal near miss cases would emerge” and was assessed based on the WHO criteria [12].

## Methods

This hospital-based prospective cohort study was conducted from November 2019 to July 2020. The study included 346 pregnant women in the third trimester (between 28 to 32 weeks) who were followed up after childbirth. A single administration of risk assessment approach was done in the third trimester.

There are seven provinces in Nepal. The study was conducted at Koshi Hospital in the Morang district, a referral hospital for Province 1. The total population in the Morang district is 1,058,985, with 27,883 expected pregnancies annually [24]. Koshi Hospital provides antenatal services to an average of 65–70 clients per day and 18,702 clients per year [25]. According to the literature, 41.0% of pregnancies in Nepal were considered high-risk in 2000 [26] and 24.6% in 2015 [27]. Nationally, only about 69% of pregnant women complete the recommended fourth antenatal visit in line with the national protocol [10], whereas, in the Morang district, 55.3% of women comply [28].

Nepal follows the WHO recommendation of four ANC visits at 4, 6, 8, and 9 months [29, 30]. Routine ANC includes iron supplementation, blood tests (hemoglobin, blood group, Rhesus factor, hepatitis B surface antigen, sexually transmitted infections), urine tests (albumin, protein, sugar), retrovirus test, tetanus-diphtheria injections, measurement of blood pressure, anthelminthic drugs, and health education regarding pregnancy [31]. The services provided are documented on antenatal cards, which remain with the pregnant women. Following childbirth, women are hospitalized for at least 12 hours for normal births and a minimum of 3 days for Cesarean sections.

The study population was all pregnant women, and the source population included pregnant women who had ANC follow-ups at Koshi Hospital. The eligible participants were pregnant women between 28 and 32 gestational weeks aged ≥18 years who were Nepalese citizens residing in the Morang district. Women more than 42 days postpartum were excluded from the study. Consecutive sampling was applied. One to two weeks before the expected date of birth, the enrolled participants were called to enquire at which hospital they planned to give birth. Hospital admissions and birth records were checked daily based on the women’s names and addresses to confirm the follow-ups.

The main outcome variable was SMM status. Risk stratification was a fixed variable. Age and mode of birth were treated as confounding variables based on previous literature [32, 33]. SMM cases were identified from the hospital records based on the presence of at least one of the WHO based criteria. The risk stratification based on color-code was done based on the presence of severe risk factors, e.g., women with the presence of one risk factor from the red color-code criteria were stratified as red color although she had risk factors from yellow and green color-code criteria.

A case report form was developed for data collection (S1 File). Each case report form included sociodemographic and economic information, history of previous pregnancies, the Malaysian antenatal risk stratification markers, and the WHO SMM criteria. The antenatal risk stratification markers were adopted from the checklist guideline for health care of mother and baby following the color code system [19].

Two research assistants with diplomas in nursing were provided with the appropriate training. Pregnant women were approached to ensure they met the inclusion criteria, and their written consent was obtained prior to their participation in the study. Using face-to-face interviews, the women’s sociodemographic and economic information were collected. The women’s ANC cards, outpatient department cards, and self-reports were used for the color-coded risk stratification. These pregnant women were followed-up after childbirth; the required information on SMM conditions was retrieved from the women’s medical records on the day of discharge.

Using Power and Sample Size Calculation software version 3.1.6, the sample size was calculated by comparing two proportions to determine the association between risk stratification and SMM, which provided the largest sample size of the two objectives. The proportion of high-risk pregnancies among women with SMM was 24%, and the proportion of women without high-risk pregnancies and SMM was 11.4% [34]. The ratio of non-severe maternal morbidity women to severe maternal morbidity women that was used was 2:1. The minimum calculated sample size was 309. After considering a 20% non-response rate, the required sample size was 370.

The data were entered, cleaned, and analyzed using IBM SPSS Statistics version 26.0. Descriptive analysis was used to determine the prevalence of high-risk pregnancies and SMM. Simple and multiple logistic regression confirmatory analyses were done to explore the association between risk stratification and SMM. The odds ratios and 95% confidence intervals were calculated, and *p* < 0.05 was considered statistically significant.

Ethical approval was obtained from the Human Research Ethics Committee Universiti Sains Malaysia (USM/JEPeM/19060356) and Nepal Health Research Council (Reg. no. 336/2019). The written consent of the women was obtained before the interviews. The hospital management was notified of women who had been color-coded as red so that they could receive the necessary attention.

## Results

A total of 370 eligible women attending antenatal clinics in their third trimester (28–32 gestation weeks) were recruited for this study. Of these, 346 completed the follow-up following childbirth, providing a response rate of 93.5%. Women lost to follow-up were those who provided the wrong mobile number or relative’s mobile number and did not give birth at the Koshi Hospital. No women died during pregnancy or childbirth, and one neonatal death was reported during the study period. The prevalence of high-risk pregnancy was 14.4% (n = 50).

About 18% of high-risk pregnant women developed SMM conditions. While about 5% of low-risk pregnant women developed SMM conditions in which seven (4.5%) had hypertensive disorders, four (3.3%) had haemorrhagic disorders, and seven (4.5%) were managed for their complications. In the present study, the majority of the women color-coded red visited the hospital with the complaint of premature contractions of the uterus (4.0%), Pre-labor rupture of membrane (2.6%), prolonged fever ≥five days (1.1%), and severe hypertension (1.1%). Nephrolithiasis and hypothyroidism were the main medical problems mentioned by the pregnant women who were receiving medication and were stratified yellow according to the color code system. The risk stratification characteristics and their distribution according to SMM status among the women in their third trimester are shown in Table 1.

**Table 1:** Risk stratification and distribution according to severe maternal morbidity status among pregnant women in their third trimester.

	non-SMM^a^ (n = 323)		SMM^a^ (n = 23)		Total (n = 346)	
Characteristics	n	(%)	n	(%)	n	(%)
***Red***					***26***	***(7*.*5)***
Premature contractions of the uterus Pre-labor rupture of membrane	13	(4.0)	1	(4.3)	14	(4.0)
Prolonged fever ≥5 days	9	(2.8)	0	(0.0)	9	(2.6)
High blood pressure ≥140/90 mmHg with the presence of symptoms	4	(1.2)	0	(0.0)	4	(1.1)
High blood pressure ≥170/110 mmHg	0	(0.0)	3	(13.0)	3	(0.9)
Pre-eclampsia (blood pressure ≥140/90 mmHg with urine albumin >1+)	0	(0.0)	1	(4.3)	1	(0.3)
Shortness of breath when doing light activities	0	(0.0)	1	(4.3)	1	(0.3)
Fetal heart rate ≤110/min at and after 26 gestational weeks	1	(0.3)	0	(0.0)	1	(0.3)
***Yellow***					***24***	***(6*.*9)***
Mother with medical problems who need hospital treatment	15	(4.6)	3	(13.0)	18	(5.2)
History of infertility before the current pregnancy	3	(0.9)	0	(0.0)	3	(0.9)
Hemoglobin ≥7 to <9 gm% or symptomatic	2	(0.6)	0	(0.0)	2	(0.6)
High blood pressure between 140/90 and 170/110 mmHg with urine albumin negative	0	(0.0)	2	(8.7)	2	(0.6)
Diabetes with insulin treatment	1	(0.3)	0	(0.0)	1	(0.3)
***Green***					***249***	***(72*.*0)***
Primigravida	182	(56.3)	17	(73.9)	199	(79.9)
Mother’s weight before pregnancy or when booking <45 kg	70	(21.7)	3	(13.0)	73	(29.3)
Birth spacing less than 2 years or over 5 years	54	(16.7)	5	(21.7)	59	(23.7)
Hemoglobin ≥9 to ≤11 gm%	54	(16.7)	1	(4.3)	55	(22.0)
Height <145 cm	30	(9.3)	2	(8.7)	32	(12.8)
Current medical problems (including psychiatric and physical defects) except diabetes and hypertension	10	(3.1)	1	(4.3)	11	(4.4)
Rh negative	7	(2.2)	0	(0.0)	7	(2.8)
Multiple pregnancies	3	(0.9)	0	(0.0)	3	(1.2)
High blood pressure (>140/90 mmHg) without urine albumin	0	(0.0)	1	(4.3)	1	(0.4)
Static body weight or decreased (per month)	1	(0.3)	0	(0.0)	1	(0.4)
*Past obstetric history*						
• Cesarean section	22	(6.8)	2	(8.7)	24	(9.6)
• Perinatal death	7	(2.2)	1	(4.3)	8	(3.2)
• Baby’s history with birth weight <2.5 kg or >4 kg	7	(2.2)	0	(0.0)	7	(2.8)
• Previous any gynecological surgery	4	(1.4)	1	(4.3)	5	(2.0)
• Third-degree perineal tear	1	(0.3)	0	(0.0)	1	(0.4)
• Instrumental birth	1	(0.3)	0	(0.0)	1	(0.4)
• Prolonged birth pains	1	(0.3)	0	(0.0)	1	(0.4)
• History of pregnancy-induced hypertension/eclampsia	0	(0.0)	1	(4.3)	1	(0.4)
***White***					***47***	***(13*.*6)***
Medical problems in a previous pregnancy	34	(10.5)	6	(26.1)	40	(11.6)
Gravida 2 to 5	33	(10.2)	1	(4.3)	34	(9.8)
Previous obstetric problems that may be repeated or affect the current pregnancy	29	(9.0)	3	(13.0)	32	(9.2)
Measured height <145 cm	30	(9.3)	2	(8.7)	32	(9.2)
Obstetric problems in the current pregnancy	16	(5.0)	1	(4.3)	17	(4.9)
Baby weight estimates between 2 kg and 3.5 kg	1	(0.3)	0	(0.0)	1	(0.3)

^a^ Severe maternal morbidity

The study found an association between risk stratification and SMM (Table 2). The high-risk pregnant women were 4.2 times more likely to develop SMM compared to the low-risk pregnant women (Table 2). Area under receiver operating characteristics curve is 0.677 (95% CI: 0.537, 0.818) and Hosmer Lemeshow test, *p*-value = 0.139.

**Table 2:** Association between risk stratification and severe maternal morbidity among pregnant women in their third trimester using multiple logistic regression confirmatory analysis.

Variables (n = 346)	Crude OR^a^	(95% CI^b^)	Adj *OR*^c^	(95% CI^b^)	Wald stat^d^ (df)^e^	*p*-value
Risk stratification						
Low-risk	1		1			
High-risk	4.22	(1.79, 10.86)	4.21	(1.68, 10.50)	9.47 (1)	0.002

^a^ Crude odds ratio

^b^ Confidence interval

^c^ Adjusted odds ratio

^d^ Wald statistics

^e^ Degree of freedom

## Discussion

Nearly 15% of women who attended the referral hospital in Nepal were identified as high-risk pregnancies. It is similar to the WHO estimates that 15% of pregnant women may need specialized obstetric care to manage complications. The percentage of women stratified as low-risk were nearly 85%; however, only 13.6% of these pregnancies were categorized into white color codes that are normal pregnancies without any medical or obstetric problems. More than two-thirds of the pregnant women were categorized into green color codes with mild medical problems in current pregnancy or with previous pregnancies complications. Women with high-risk pregnancies were nearly four times more likely to develop SMM later in childbirth than low-risk pregnancies. The positive predictive value of this risk stratification tool was 67.7% in predicting SMM.

Worldwide, 10%–30% of pregnancies are estimated to be “at-risk” [35–37]. The prevalence of high-risk pregnancies in the present study was 14.4%, within the global range of at-risk pregnancies. A cross-sectional study conducted in two referral hospitals in Kelantan, Malaysia, used a similar color-coded antenatal risk stratification. It reported a prevalence of 11.4% of high-risk pregnancies [34], similar to our findings. However, a retrospective cohort study conducted in primary health care clinics in Selangor, Malaysia, using the same risk approach reported a prevalence of 28% of high-risk pregnancies [38]. It is almost double the current study indicating a possible differencein thelevel of health care, i.e., primary health clinics vs. referral hospitals.

Studies in different countries had used different risk stratification criteria and tools; thus, the magnitudes of high-risk pregnancies differ across countries. A risk stratification approach that classifies higher proportions of women as high-risk is not feasible for recommendation to use in a public health system. Different diagnostic criteria were used; therefore, true comparisons were not feasible across studies. Risk assessments done in the third trimester or at the bedside have shown lower percentages of high-risk pregnancies than those done at the time of admission. A study conducted in 2002 in the United Kingdom compared the Knox scoring system to the Chard *et al*. risk scoring system. The prevalence of high-risk pregnancies on admission for the Knox scoring system was 11.7% vs. 48.9% for the Chard *et al*. risk scoring system, and at 36 weeks’ gestation, the prevalence using the same two systems was 1.4% vs. 38%, respectively [39]. The prevalence of high-risk pregnancies in New Zealand was 16% in 1993 [40] and 37% in 1990 [41]; the first study used the Knox scoring system while the second used a self-developed scoring system. In the United States, a 2.3% prevalence of high-risk pregnancies was observed in 2018 using the obstetric comorbidity index in which the scoring is done at the time of labor [42]. The prevalence of high-risk pregnancies in developed countries differs based on the gestational week and scoring systems used.

In the Middle East, using a modified Morrison and Olsen risk assessment tool, the highest prevalence was in Egypt (63.8%) in 2005 [43] and Iran (63.5%) in 2012 [44]. In Turkey, the prevalence of high-risk pregnancies at the time of booking using the Knox scoring system was 50.4% in 2013 [45], while the Turkey Demographic Health Survey estimate was 69.4% in 2004 [46]. Using a modified Morrison and Oslen risk scoring tool in Saudi Arabia, the reported prevalence of high-risk pregnancies was 63.3% in 2014 [47]. However, using the Coopland *et al*. scoring tool, the same country’s prevalence was 45% in 1989 [48]. There are two possible reasons for these very high prevalence. First, the diagnostic criteria are sensitive to the presence of even a single risk factor, which affects the overall sensitivity of the tools. Second, the predictive accuracy of the scoring system depends on the burden of outcomes, such as very high fertility rates in the population [39].

The Malaysian antenatal risk stratification approach, which screens pregnant women based on antepartum risk factors, was used in the current study. Other risk assessment tools use either antepartum risk factors or intrapartum risk factors, or both [27, 49–51]. Some risk assessment tools collect neonatal risk factors, as well [51]. The Malaysian risk stratification approach has 15 risk factors in the red category, 14 in the yellow category, 32 in the green category, and 9 in the white category. The current study took around 7–10 minutes to assess each woman’s risk factors using antenatal card records and self-reporting. A risk assessment tool developed by Hobel *et al*. [50] had 49 antepartum and 36 intrapartum factors, and 5 minutes were required to complete the form. In contrast, a simple risk scoring system used in Nepal had 28 antepartum and 16 intrapartum factors [27]. An obstetric risk scoring tool used in Australia had 53 antepartum, 41 intrapartum, and 37 neonatal factors [51].

Researchers have examined the association between risk stratification and SMM, but such studies have mainly been carried out in high-income countries. The present study’s findings showed a significant association between risk stratification and SMM after adjusting for the confounders age and mode of birth. A systematic review of SMM found that the most common preventable factors in SMM cases were provider-related, specifically, a failure to identify “high-risk” status and delays in diagnosis and treatment [11]. In Brazil, the relative risk of MNM is 4.5 times greater among high-risk women than low-risk women [15], which supports the current study’s findings. In a case-control study in Malaysia, 22.5% of SMM cases belonged to high-risk pregnancies. Whereas, among women without SMM conditions, 11.4% belong to the high-risk pregnancies [34]. Studies have shown a significant association between risk stratification and maternal morbidities, but the WHO-based SMM criteria were not used [7, 52, 53].

Studies using risk scoring tools have found a significant association between high-risk pregnancies and obstetric complications. A retrospective study of birth records in Canada showed that adverse pregnancy outcomes increased with increased antepartum risk scores [22]. Berglund *et al*. also found that the relative risk of pregnancy complications increased significantly among women who were found to have high-risk pregnancies [53]. Meanwhile, Morrison *et al*. in 1980 and Kolluru *et al*. in 2016 both showed that the need for surgical interventions significantly increased among women with high-risk scores [7, 54].

The presence of comorbidities among pregnant women has significantly increased the risk progression to SMM [55]. The obstetric comorbidity index is used in the United States, but it is applied at the time of labor. A considerable volume of evidence has been gathered from studies in the United States, showing that high-risk pregnancies detected using obstetric comorbidity index scores are associated with high SMM rates [42, 56–58]. Three studies conducted in Canada, Denmark, and the United States each used the obstetric comorbidity index retrospectively in huge sample sizes. All had consistent findings of an increased risk of severe maternal outcomes with every one-point increase in the obstetric comorbidity index [59–61]. A study conducted in Canada found that the presence of major preexisting conditions increased women’s risk of SMM six-fold [62]. In contrast, according to a study in the United States, this risk increased two-fold [63]. These findings were all consistent with those of the current study. A possible explanation for these significant associations is likely that the risk factors included in the risk assessment tools to identify high-risk pregnancies were determinants derived from an improved understanding of their positive association with either maternal morbidity or mortality based on previous studies [64]. These risk factors have thus been included in scoring tools because it has been shown that they increase the relative risk of adverse maternal complications [64]. The use of risk scoring systems to predict SMM therefore, shows significant potential.

The area under the receiver operating characteristic curve in predicting SMM using the risk stratification approach was 68% in the current study. In the United States, the areas under the curve in studies that used comorbidity index to predict SMMwere 78% [56], 65% [60], and 70% [65]. These were similar to that of the current study. Studies in Canada and Denmark using the same obstetric comorbidity index showed areas under the curve of 64% [59] and 70% [61]. In Turkey, where the Knox scoring system was used, the area under the curve on admission was 65%, while at the onset of labor, it was 94% [45].

Although the number of the high-risk pregnancies was proportionally smaller, the overall accuracy in predicting SMM using the present study’s risk assessment approach was 18%. The positive predictive value of maternal morbidity outcomes using a modified early obstetric warning system in the United Kingdom was 39% [66]. The sensitivity of maternal early warning systems is generally acceptable; however, the positive predictive values have been consistently low in predicting maternal morbidity [67, 68].

With their continual modifications, there is an ongoing debate on the use of risk scoring tools. Few studies have entirely dismissed the “at-risk” concept, stating that “there is no pregnancy without risk” [3, 69, 70]. Still, they have questioned the accuracy of the scoring tools and indicated that unnecessary medical interventions are problematic in false-positive cases [71]. A Cochrane review on risk scoring systems mentioned that the system’s measures need to be relatively high for the correct classification of high-risk pregnancies [72]. One study concluded that many of the risk factors for high-risk pregnancies have only shown statistical associations with adverse outcomes but no evidence of actual causation [5]. A high-risk pregnancy is identified based on the presence of risk factors. Although the relative risk of most of these risk factors is high, most high-risk pregnant women do not experience adverse outcomes, while low-risk women may end up with adverse outcomes [73]. It may be because most risk factors, even if they are strongly associated with negative outcomes in populations, do not predict adverse outcomes very well at an individual level [71].

A risk assessment tool’s effectiveness can be determined by whether it appropriately identifies women likely to have poor pregnancy outcomes [74]. Complications during the intrapartum period can be due to various factors, especially due to three delays [75–77], mainly the third delay due to lack of adequate infrastructure for care and emergency obstetric interventions unavailable hospital [78, 79]. A risk assessment tool is not a substitute for clinical judgement [22], but it can flag women who need clinical attention. In a prospective study conducted in the United States, more than 80% of all perinatal deaths occurred among one-quarter of high-risk pregnant women [80]. Conversely, risk assessment scores predict 73% of normal outcome events in low-risk women [54]. Although smaller in proportion, the occurrence of maternal and neonatal adverse outcomes is higher among high-risk pregnancies.

Heins *et al*. conducted a quasi-experimental study in which not all but some women with high-risk pregnancies and low incomes were allocated to the intervention group for which medical care was offered [81]. Interestingly, despite an increasing perinatal death rate as the high-risk scores increased, there was a significant association with reduced perinatal mortality in the intervention group comprising women with high-risk pregnancies. Similarly, a study in the United States was conducted in high- and low-acuity hospitals to assess the comorbidity index’s effects on SMM risk [82]. A clinical pathway of maternal early warning triggers for four maternal morbidities (i.e., sepsis, cardiovascular dysfunction, severe preeclampsia/hypertension, and hemorrhage) expedited assessments and treatment for the women in the intervention group, which significantly reduced SMM. The findings showed that high-risk women have a higher risk of SMM at low-acuity hospitals. Early identification of at-risk populations is mainly undertaken so that effective interventions can be provided. These two studies’ findings support the belief that interventions in high-risk pregnancies could reduce adverse maternal morbidity outcomes [81].

### Strengths and limitations

This is one of the first studies in Nepal to explore the association between risk stratification and SMM. This study provides an estimate of high-risk pregnancies that may help management at tertiary hospitals in their preparations for such cases. Nevertheless, the current study had a few limitations that need to be considered before generalizing its findings.

This study was conducted at a single tertiary hospital, which limits the generalization of its findings at a population level. The risk assessment was also only administered once. In contrast, assessments are conducted at each visit in Malaysia. This study did not follow this protocol exactly because of Nepal’s lack of an established referral system. Accordingly, not all the risk assessment system risk factors were assessed earlier than 28 weeks or beyond 32 weeks of gestation. Furthermore, SMM criteria were used rather than MNM criteria because most of the laboratory-based criteria and management-based criteria could not be applied in low resource settings.

### Recommendations

Antenatal risk screening has an advantage over routine ANC-only care, so its use is encouraged. Earlier identification and the routine use of the risk assessment system rather than its single administration will be beneficial as the health of pregnant women tends to fluctuate due to physiological changes during pregnancy. Risk stratification aims to ensure that high-risk women receive timely and appropriate additional care to prevent poor maternal outcomes. A protocol for managing risk conditions and referral systems will be required if the risk assessment approach is introduced. Future studies can utilize this risk stratification approach at peripheral health institutions to study its feasibility within the existing public health system.

## Conclusions

The prevalence of high-risk pregnancies assessed using the color-coded antenatal risk assessment approach in the present study was 14.4%. A significant association was found between risk stratification and SMM. The risk assessment approach used in this study is a useful tool for identifying high-risk women at risk of developing adverse obstetric outcomes. The overall accuracy in predicting SMM using the risk assessment approach in the present study was smaller but still significant for high-risk pregnancies. Although proportionally smaller, the chances of occurrence of adverse maternal outcomes were higher among high-risk pregnancies. Therefore, the use of the color-coded risk scoring system shows potential to use and is recommended in future studies at the primary health care level in Nepal. Although unpredictable SMM events occur among low-risk pregnant women in smaller percentages, all women require vigilance and quality obstetrics care. The women with high-risk pregnancies are suggested to be referred for extensive care and management.

## Supporting information

S1 File Case report form. (DOCX)
