# Supplementary material for: High-risk pregnancies and their association with severe maternal morbidity in Nepal: A prospective cohort study
Source: PLoS One. 2020 Dec 28;15(12):e0244072. doi: 10.1371/journal.pone.0244072 (PMC7769286; doi:10.1371/journal.pone.0244072)
Supplement: S1 File — (DOCX) [file pone.0244072.s001.docx]

***Research Title: Severe Maternal and Neonatal Morbidity Status and Quality of Care in Morang District, Nepal***

Case Report Form for Phase I

*Participant: Pregnant woman followed up till their childbirth*

Date: _____/______/______

Please tick √ for answer given by the participant.

Inclusion criteria:

Woman aged ≥18 years  Yes No

28 to 32 weeks of pregnancy Yes No

Exclusion criteria:

> 42 days of termination of pregnancy Yes No

**Section A: Respondent**

| ID No. _______ Unique ID Code. ________________ | |
| --- | --- |
| Caste: _________________________ |  |
| Gestation weeks: _________ | EDD: ________________ |
| Date of admission: _______________ | Date of discharge: _______________ |
| Duration of hospitalization: __________ (Days) | Date of birth: ___________________ |
| Address: ___________________ Tole Name: _______________ Ward no: _______________  Nagarpalika  Gaunpalika | |
| Phone number (mobile) : ___________________________ self husband | |

**Medical records review with pregnant woman**

**Section A: Risk stratification**

| 1. **Red color code** |
| --- |
| Eclampsia  Preeclampsia (blood pressure high with urine albumin) i.e. BP≥140/90mmHg with urine albumin >1+  High blood pressure ≥170/110mmHg  High blood pressure >140/90mmHg with presence of symptoms  Heart disease during pregnancy with sign and symptoms (shortness of breath, pounding)  Shortness of breath when doing light activities (activities such as broomstick, dishwashing)  Uncontrolled Diabetic mother with the presence of urine ketone  Antepartum bleeding (including miscarriage)  *Fetal heartbeat abnormal* FHR ≤110/min at and after 26/52 FHR >160/min after 34/52 (heartbeat may be high if premature)  Anemia symptoms at any gestation or Hb ≤7gm%  Premature contraction of the uterus  Leakege of amniotic fluid without contraction  Severe asthma attacks  Convulsions  Prolonged fever ≥5 days |
| 1. **Yellow color code** |
| Mothers is HIV positive  Mother is Hepatitis B positive  Mother with Tuberculosis /Malaria/Syphilis  High blood pressure <170/110 to >140/90 mmHg with urine albumin negative  Diabetic mother (with insulin treatment)  Reduced fetal movement at ≥32 weeks gestation  Postdate exceeds more than 7 days then EDD  Mother with medical problems who need hospital treatment  Mother is involved in medical legal issue  Single mothers or teenage mothers (<19 years)  Hemoglobin < 9 to 7gm% or symptomatic  Stable placenta previa - no bleeding  Maternal pyrexia >38˚C or >3 days  History of infertility before current pregnancy  Heart disease without symptoms  Drug addiction /smoking |
| 1. **Green color code** |
| Rh Negative  Mother's weight before pregnancy or when booking <45kg  Current medical problems (including psychiatrists and physical impairment) except diabetes and hypertension  Previous any gynecological surgery  Uncertain Last Normal Menstrual Period  3 times history of sequential miscarriage |
| Past obstetric history: |
| Caesarean section  History of past PIH /eclampsia/diabetes  Perinatal death  Have a baby's history with less birth weight of 2.5kg or more than 4 kg  History of 3rd degree perineal tear  Retained placenta  Postpartum bleeding  Instrumental birth  Prolonged delivery pains |
| Multiple pregnancy  High blood pressure (140/90mmHg) without urine albumin  Hemoglobin ≤11 to 9gm%  Glucosuria 2 times  Urine has albumin ≥1+  Weight gain more than 2kg in a week  Maternal weight before pregnancy or during booking over 80 kg  Uterus height (SFH) or bigger or smaller than pregnancy date  Inverted/oblique/transverse with none birthmark at 36 weeks of pregnancy  Head not engaged at more than 37 weeks for primi-gravida  Mothers GDM (diet control)  Static body weight or decreased in weight (in period per month)  Mother is >40 years old  Primi-gravida  Gravida 6 and up  Birth spacing less than 2 years or more than 5 years  Height less than 145cm |
| 1. **White code color** |
| Gravida 2-5  No previous obstetric problems which may repeat or effect on current pregnancy  No medical problems before  No obstetric problems in current pregnancy  Measuring height is over 145cm  Mother is over 18 years of age and less than 40 years old  Mother is married and has family support  POA (Premature Ovarian Aging) >37 weeks or <41 weeks  Estimated baby weight is >2kg and <3.5kg |

**Medical records review for pregnant women**

**Section B: Current obstetric conditions**

| SN | Current obstetric conditions | Options |
| --- | --- | --- |
| 101 | Total number of antenatal visits | ________ times |
| 102 | Problems detected during antenatal visits | ____________________________ (specify)  ____________________________ (specify)  ____________________________ (specify)  ____________________________ (specify) |
| 103 | Color codes | Red  Yellow  Green  White |
| 104 | Mode of delivery | Spontaneous vaginal delivery  Assisted vaginal delivery  Emergency caesarean section  Elective caesarean section  Others ____________ (specify) |

*Note: 101 & 104 data will be obtained after childbirth*

**Section C: Clinical parameters**

| SN | Clinical parameters | Options |
| --- | --- | --- |
| 201 | Pre-pregnancy weight | _______ kg |
| 202 | Pre-pregnancy height | _______ cm |
| 203 | Pre-pregnancy BMI | _______ kg/m^2^ |
| 204 | Blood pressure | Systolic ________ mm/Hg  Diastolic ________ mm/Hg |
| 205 | Haemoglobin | ________________ g/dL |

**Medical records review at childbirth after matching the unique identification code**

**Section D: Severe maternal morbidity status**

| **Haemorrhagic disorders** |
| --- |
| Abruptio placentae  Placenta accreta/increta/percreta  Ectopic pregnancy  Postpartum haemorrhage  Ruptured uterus |
| **Hypertensive disorders** |
| Severe pre-eclampsia  Eclampsia  Severe hypertension  Hypertensive encephalopathy  HELLP (haemolysis, elevated liver enzymes, low platelet count) syndrome |
| **Other systemic disorders** |
| Endometritis  Pulmonary oedema  Respiratory failure  Seizures  Sepsis  Shock  Thrombocytopenia (< 100,000)  Thyroid crisis |
| **Severe management indicators** |
| Blood transfusion  Central venous access  Hysterectomy  Intensive care unit admission  Prolonged hospital stay (> 7 postpartum days)  Intubation not related to anaesthetic procedure  Return to operating room  Laparotomy (includes hysterectomy, excludes caesarean section) |

**Face to face interview with pregnant woman**

**Section E: Sociodemographic data**

| SN | Questions | Options |
| --- | --- | --- |
| 301 | Ethnicity | Brahmin/Chettri  Terai/Madhesi other castes  Dalits  Newar  Janajati  Muslim  Others |
| 302 | Religion | Hindu  Buddhist  Muslim  Kirat  Christian  Other |
| 303 | Woman highest education achieved | _________ passed grade |
| 304 | Husband highest education achieved | _________ passed grade |
| 305 | Woman occupation | Housewife  Self-employed  Professional/managerial  Agriculture  Unskilled manual  Others ________________________ specify |
| 306 | Husband occupation | Professional technical/managerial  Clerical  Sales and services  Skilled manual  Unskilled manual  Agriculture  Others _________________________ specify |
| 307 | Husband smoke | Yes ______________number of sticks/day  No |
| 308 | Age | _____years |
| 309 | Age at marriage | _____ years |
| 310 | Years of marriage | _____ years |

**Section F: Wealth index**

| SN | Questions | Options |
| --- | --- | --- |
| 401 | Does your household have a television? | Yes  No |
| 402 | Does your household have a cupboard? | Yes  No |
| 403 | Does your household have a table? | Yes  No |
| 404 | Does your household have a fan? | Yes  No |
| 405 | What is the main material of the floor of your dwelling? | Earth/sand  Other |
| 406 | What is the main material of the exterior walls of your dwelling? | Cement  Other |
| 407 | What is the main material of the roof of your dwelling? | Cement  Other |
| 408 | Why type of fuel does your household mainly use for cooking? | LPG  Wood  Other |

**Section G: Past obstetric history**

| SN | Past obstetric history | Options |
| --- | --- | --- |
| 501 | Number of children | ______ None ________ Boys ______ Girls |
| 502 | Birth spacing in the last pregnancy | ________ months |
| 503 | Mode of delivery for the last pregnancy | Emergency caesarean section  Elective caesarean section  Assisted vaginal delivery  Spontaneous vaginal delivery |
| 504 | Complications in the previous pregnancy | Yes, specify  _________________________________________________  _________________________________________________  _________________________________________________  No |
| 505 | Number of abortions | Yes, ___________ times  No |
